# Supplementary material for: Cellulose-Based Hectocycle Nanopolymers: Synthesis, Molecular Docking and Adsorption of Difenoconazole from Aqueous Medium
Source: Int J Mol Sci. 2021 Jun 4;22(11):6090. doi: 10.3390/ijms22116090 (PMC8201209; doi:10.3390/ijms22116090)
Supplement: Supplementary file 1 [file ijms-22-06090-s001.zip › ijms-1234420-supplementary.pdf]

Supplementary Material

Cellulose-Based Hectocycle Nanopolymers: Synthesis, Molecular Docking and Adsorption of Difenoconazole from Aqueous Medium

Bayan Khalaf [1], Othman Hamed [1,*], Shehdeh Jodeh [1,*], Roland Bol [2], Ghadir Hanbali [1], Zaki Safi [3], Omar Dagdag [4], Avni Berisha [5,6] and Subhi Samhan [7]

1 Chemistry Department, Faculty of Science, An-Najah National University, P.O. Box 7, Nablus, Palestine.
2 Institute of Bio and Geosciences, Agrosphere (IBG-3), Forschungszentrum Jülich GmbH, 52425 Jülich, Germany.
3 Al Azhar University-Gaza, Chemistry Department, Faculty of Science, P.O Box 1277, Gaza, Palestine
4 Laboratory of Industrial Technologies and Services (LITS), Department of Process Engineering, Height School of Technology, Sidi Mohammed Ben Abdallah University, P.O. Box 2427, 30000, Fez, Morocco.
5 Department of Chemistry, Faculty of Natural and Mathematics Science, University of Prishtina, 10000 Prishtina, Kosovo.
6 Materials Science-Nanochemistry Research Group, NanoAlb-Unit of Albanian Nanoscience and Nanotechnology, 1000 Tirana, Albania.
7 Research and Development Center, Palestine Water Authority (PWA), Ramallah, Palestine.
* Correspondence: ohamed@najah.edu, Tel: +970-594-466-271 (O.H.); sjodeh@najah.edu, Tel: +970-594-466-271 (S.J.)

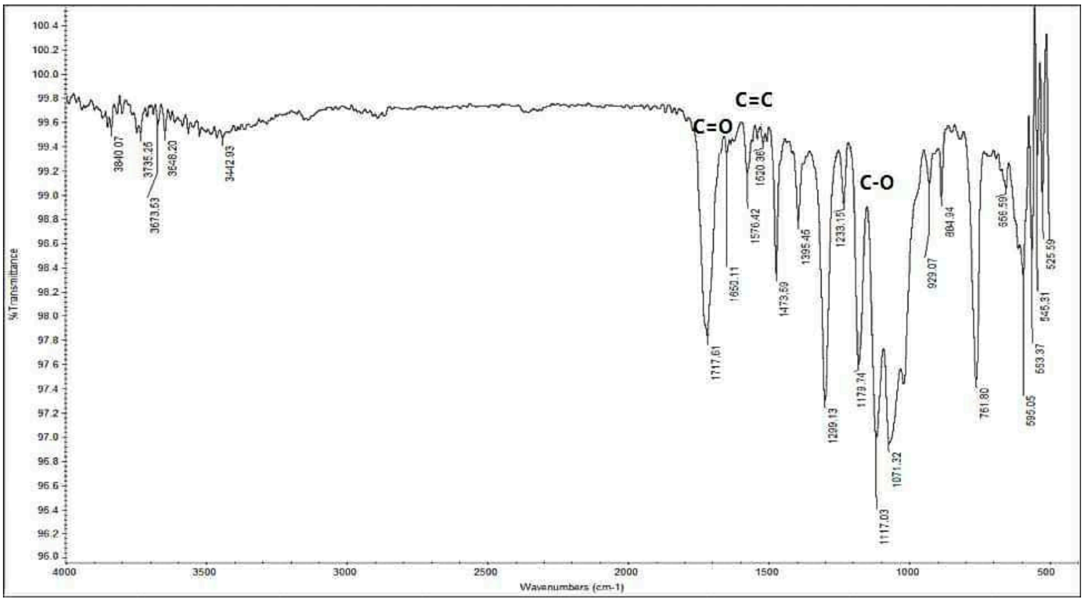

Figure S1. FT-IR analysis for Cell-D.

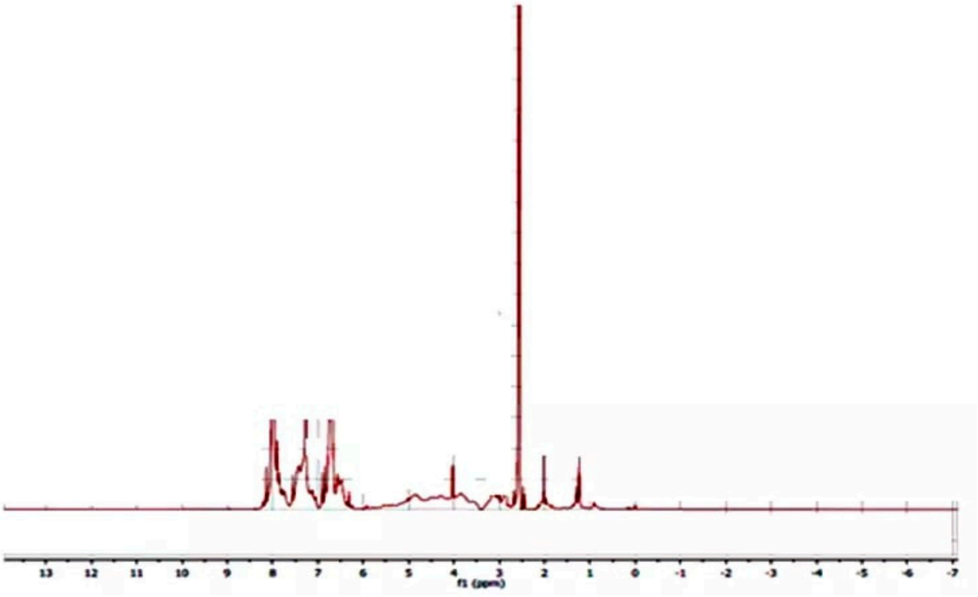

Figure S2. H^1 NMR analysis for Cell-D dissolved in DMSO-d_6.

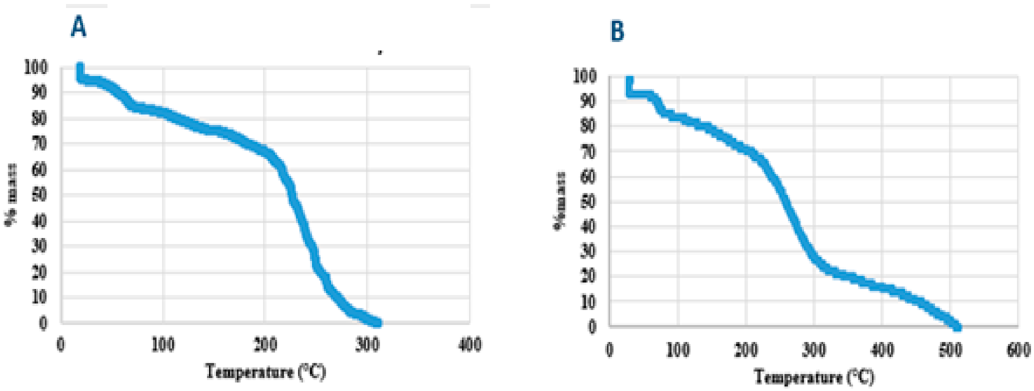

Figure S3. TGA analysis for A: Cell-D and B: Cell-X.

Table S1. Parameters of Langmuir and Freundlich isotherms for Difenoconazole by Cell-X and Cell-D.

**Nanopolymer**	**Langmuir Isotherm**		**Freundlich Isotherm**	
	**Q_o (mg/g)**	**b (L/mg)**	**K_F (mg/g)**	**n (g/L)**
Cell-D	5.893	2.173	9.270	−4.016
Cell-X	5.447	1.708	9.311	−3.618

Table S2. Pseudo-first-order, pseudo-second-order and intra-particle-diffusion kinetic adsorption parameters of Difenoconazole removal by Cell-X or Cell-D.

Nanopolymer	Pseudo First-Order Kinetics		q_{exp} (mg/g)	Pseudo Second-Order Kinetics		Intra-Particle Diffusion Kinetics	
	q_e (mg/g)	K_1 ($mg.g^{-1}.min^{-1}$)	8.524	q_e (mg/g)	K_2 ($g.mg^{-1}.min^{-1}$)	C (mg/g)	K_p ($mg.g^{-1}.min^{-0.5}$)
Cell-D	1.764	7.83×10^{-3}	8.773	8.849	0.224	7.8276	0.1483
Cel-X	2.016	8.98×10^{-3}	8.745	8.834	0.1396	7.5422	0.1746

Table S3. Thermodynamic parameters of Difenoconazole pesticide adsorption by Cell-X or Cell-D.

Nanopolymer	ΔH (kJ)	ΔG ° (kJ)	ΔS (J/K)
Cell-D	−25.442	−5.312	−68.667
Cell-X	−24.554	−4.013	−70.071
